# Renal impairment as a risk factor for trifluridine/tipiracil-induced adverse events in metastatic colorectal cancer patients from the REGOTAS study

**DOI:** 10.1038/s41598-023-45244-7

**Published:** 2023-10-20

**Authors:** Mamiko Shiroyama, Shota Fukuoka, Toshiki Masuishi, Atsuo Takashima, Yosuke Kumekawa, Takeshi Kajiwara, Kentaro Yamazaki, Yasuhiro Shimada, Taito Esaki, Akitaka Makiyama, Toshikazu Moriwaki

**Affiliations:** 1 Department of Gastroenterology, Faculty of Medicine, University of Tsukuba, Tennodai 1-1-1, Tsukuba City, Ibaraki 305-8575 Japan; 2 Division of Cancer Immunology, Exploratory Oncology Research and Clinical Trial Center, National Cancer Center, Kashiwa city, Chiba Japan; 3 Department of Clinical Oncology, Aichi Cancer Center Hospital, Nagoya City, Aichi Japan; 4 Gastrointestinal Medical Oncology Division, National Cancer Center Hospital, Chuo-ku, Tokyo Japan; 5 Department of Gastroenterology, Saitama Cancer Center, Kitaadachi-gun, Saitama Japan; 6 Department of Gastrointestinal Medical Oncology, National Hospital Organization Shikoku Cancer Center, Matsuyama city, Ehime Japan; 7 Division of Gastrointestinal Oncology, Shizuoka Cancer Center, Sunto-gun, Shizuoka Japan; 8 Clinical Oncology Division, Kochi Health Sciences Center, Kochi city, Kochi Japan; 9 Department of Gastrointestinal and Medical Oncology, National Hospital Organization Kyushu Cancer Center, Fukuoka city, Fukuoka Japan; 10 Department of Hematology/Oncology, Japan Community Healthcare Organization Kyushu Hospital, Kitakyushu city, Kitakyushu Japan

**Keywords:** Cancer therapy, Colorectal cancer, Gastrointestinal cancer

## Abstract

Renal impairment may be associated with an increased risk of hematologic events (AEs) in patients undergoing treatment with trifluridine/tipiracil (FTD/TPI). This study aimed to investigate the specific types of AEs linked to renal impairment in patients with metastatic colorectal cancer (mCRC) receiving FTD/TPI, using real-world data. Among the patients included in the REGOTAS study (a retrospective study of FTD/TPI versus regorafenib), those treated with FTD/TPI were evaluated. Creatinine clearance values of < 30, 30–60, 60–90, and > 90 mL/min were defined as severe, moderate, mild renal impairment, and normal renal function, respectively. Renal impairment was analyzed as a risk factor for grade 3 or higher AEs using a logistic regression model. Overall survival (OS) and progression-free survival (PFS) based on renal impairment were evaluated. A total of 309 patients were included in the analysis, with 124, 130, and 55 patients divided into the normal, mild, and moderate-to-severe groups, respectively. The risk of grade 3 or higher neutropenia was significantly higher in the moderate-to-severe group (odds ratio 3.47; 95% confidence interval 1.45–8.30; *P* = 0.005), but there was no significant increase in the risk of non-hematologic AEs in any of the groups. The OS and PFS of patients in the mild and moderate-to-severe groups were comparable to those in the normal group. Patients with mCRC and moderate/severe renal impairment receiving FTD/TPI therapy may develop severe neutropenia; however, FTD/TPI remains a viable treatment option due to its clinical benefit.

## Introduction

Trifluridine/tipiracil (FTD/TPI) is mainly used for treating colorectal cancer. In a phase III study (RECOURSE), FTD/TPI improved survival compared to the placebo in patients with metastatic colorectal cancer (mCRC) refractory to oxaliplatin, irinotecan, and fluoropyrimidine (median, 7.1 months vs. 5.3 months; hazard ratio [HR] 0.68, 95% confidence interval [CI] 0.58–0.81, *P* < 0.001), and it eventually became the standard later-line chemotherapy^1^.

FTD/TPI is an oral nucleotide antineoplastic agent consisting of FTD and TPI in a molar ratio of 1:0.5^2^. FTD is an active cytotoxic component of the drug that directly incorporates into the DNA strand, causing DNA dysfunction and inducing cell growth suppression and apoptosis. TPI specifically inhibits thymidine phosphorylase, which degrades FTD and increases its bioavailability. FTD is metabolized intrahepatically by thymidine phosphorylase, whereas TPI is excreted by the kidneys. Therefore, FTD/TPI has a safety profile when administered to patients with renal or liver impairment owing to its metabolic mechanism. In a phase I study evaluating the safety of FTD/TPI in advanced solid cancer patients with mild-to-moderate renal impairment, although the recommended dose was the same as that in patients with normal renal function, hematologic adverse events (AEs), such as severe anemia and neutropenia, tended to develop in the target group^3^. In a post-marketing surveillance study on the use of FTD/TPI by Japanese patients with mCRC, renal impairment was found to be an independent risk factor for the onset of grade 3 or higher hematologic AEs, whereas hepatic impairment was not^4^. To our knowledge, previous studies have not determined the specific types of AEs caused by the risk factor of renal impairment. Therefore, this study aimed to clarify the types of AEs associated with renal impairment in patients with mCRC on FTD/TPI therapy.

## Patients and methods

### Patients

This study used data from a retrospective study that compared regorafenib and FTD/TPI in patients with mCRC refractory to standard chemotherapy (regorafenib versus FTD/TPI as salvage-line in patients with mCRC refractory to standard chemotherapies [REGOTAS] study, registration No. UMIN000020416)^5^. The REGOTAS was a retrospective study conducted between June 2014 and September 2015. Data were collected from the 24 participating institutions of the Japanese Society for Cancer of the Colon and Rectum (JSCCR). Among patients in the FTD/TPI group, those whose renal function was reduced at the initial dose and those whose renal function had not been evaluated before the initial treatment were excluded. This study was approved by the Ethics Committee of the JSCCR, National Cancer Center, Aichi Cancer Center, Saitama Cancer Center, Shikoku Cancer Center, Shizuoka Cancer Center, Kochi Health Sciences Center, National Kyushu Cancer Center, Japan Community Healthcare Organization Kyushu Hospital, Chiba Cancer Center, Kobe City Medical Center General Hospital, Yamagata Prefectural Central Hospital, Osaka International Cancer Institute, Tokyo Medical University, Tokyo Medical and Dental University, Saga University, Kyushu University, Hokkaido University Hospital, Kindai University, Kobe University, Kagawa University, Osaka University, National Defense Medical College Hospital, and University of Tsukuba Hospital and was conducted in accordance with the guidelines of the Declaration of Helsinki. This was an opt-out study, and the requirement for informed consent from the study subjects was waived by the JSCCR, National Cancer Center, Aichi Cancer Center, Saitama Cancer Center, Shikoku Cancer Center, Shizuoka Cancer Center, Kochi Health Sciences Center, National Kyushu Cancer Center, Japan Community Healthcare Organization Kyushu Hospital, Chiba Cancer Center, Kobe City Medical Center General Hospital, Yamagata Prefectural Central Hospital, Osaka International Cancer Institute, Tokyo Medical University, Tokyo Medical and Dental University, Saga University, Kyushu University, Hokkaido University Hospital, Kindai University, Kobe University, Kagawa University, Osaka University, National Defense Medical College Hospital, and University of Tsukuba Hospital due to the retrospective study design.

### Data collection

The following data were collected before treatment initiation: age, sex, body surface area, serum creatinine level, Eastern Clinical Oncology Group performance status, primary lesion site, primary tumor resection, histological type, *RAS* mutation status, metastatic lesions, number of metastatic organs, duration from the initiation of first-line chemotherapy, prior chemotherapy, and carcinoembryonic antigen level. The hematologic AEs were neutropenia, anemia, and thrombocytopenia, whereas the non-hematologic AEs were fatigue, anorexia, diarrhea, stomatitis, infection, febrile neutropenia, nausea, liver dysfunction, interstitial pneumonia, and skin disorders.

### Statistical analysis

The primary endpoint in this study was grade 3 or higher AEs according to renal impairment. The secondary endpoints were progression-free survival (PFS) and overall survival (OS) according to renal impairment. The degree of renal impairment was assessed based on creatinine clearance (Ccr) values calculated using the Cockcroft–Gault method. Patients with Ccr values of < 30 mL/min were classified as having severe renal impairment, those with 30–60 mL/min as moderate, 60–90 mL/min as mild, and > 90 mL/min as normal renal function. Patients with normal renal function (normal group) were used as controls and were compared with patients with mild renal impairment (mild group) and patients with moderate or severe renal impairment (moderate-to-severe group). Grade 3 or higher AEs were assessed according to the Common Terminology Criteria for Adverse Events (CTCAE), version 4.0, a standardized system for grading the severity of AEs^6^. We analyzed AEs induced by renal impairment using a multivariate logistic regression model. The odds ratios (ORs) of AEs were adjusted for patient background factors that exhibited significant differences (*P* < 0.1) between the mild or moderate-to-severe groups and the normal group. Overall survival was defined as the time from the start of FTD/TPI treatment until death from any cause. PFS was defined as the period from the start of FTD/TPI treatment to disease progression or death from any cause. IBM Statistics SPSS (version 25.0; SPSS Inc., Chicago, IL, USA) was used for the statistical analyses. Statistical significance was set at *P* < 0.05.

## Results

### Patients

**Figure 1:**
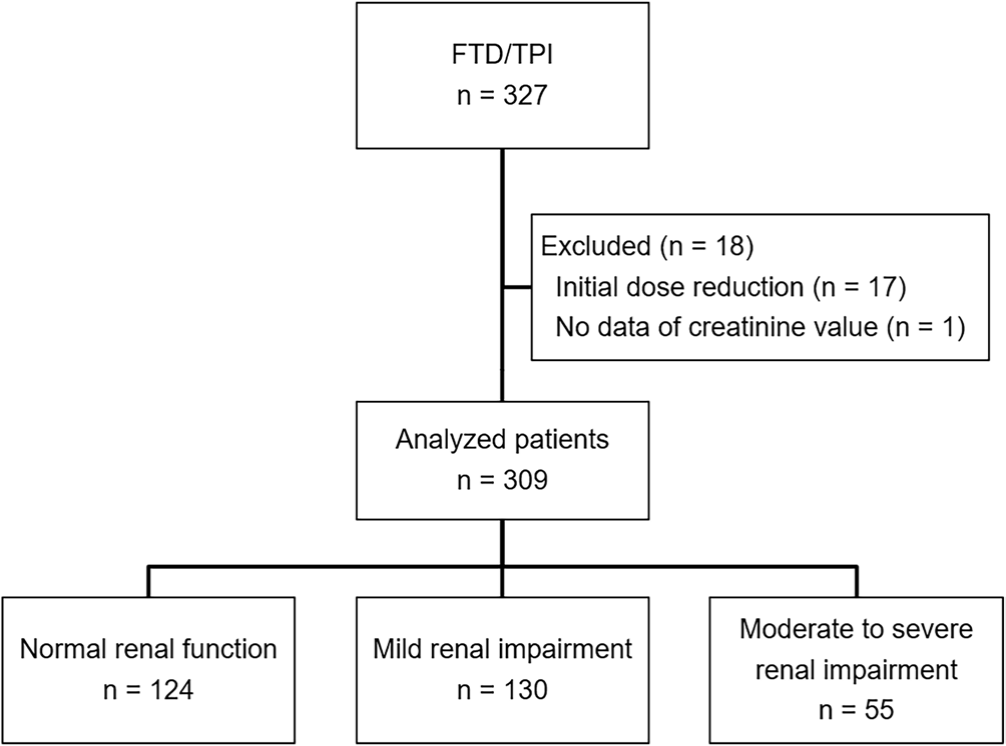
Patient selection flowchart. FTD/TPI, trifluridine/tipiracil.

Among the enrolled 327 patients in the FTD/TPI group in the REGOTAS study, 309 were included (Fig. 1); 124, 130, and 55 patients were divided into the normal, mild, and moderate-to-severe groups, respectively. Of these patients, four had severe renal impairment. The background details of the patients are described in Table 1. Compared to the normal group, the mild and moderate-to-severe groups were older and had median body surface area of less than 1.69 m^2^ (*P* < 0.001). In the mild group, well and moderately differentiated adenocarcinomas were common (*P* = 0.02), whereas bone metastasis was less common (*P* = 0.04). Liver metastases were less common in the moderate-to-severe group (*P* = 0.009).

**Table 1:** Patient characteristics.

	Normal (n = 124)		Mild (n = 130)		Moderate to severe (n = 55)		*P* value (vs normal)	
	n (%)		n (%)		n (%)		Mild	Moderate to severe
Age								
Median (range), years	57	(29–79)	66	(39–86)	72	(45–82)	< 0.001*	< 0.001*
≥ 65 years	22	(18)	81	(62)	44	(80)	< 0.001	< 0.001
Sex							0.77	0.28
Male	76	(61)	82	(63)	29	(53)		
Female	48	(39)	48	(37)	26	(47)		
ECOG PS							0.71	0.32
0	54	(44)	50	(38)	19	(34)		
1	62	(50)	71	(55)	34	(62)		
2	8	(6)	9	(7)	2	(4)		
BSA								
Median (range), m^2^	1.69	(1.28–2.13)	1.55	(1.20–2.15)	1.44	(1.06–1.81)	< 0.001*	< 0.001*
< 1.07 m^2^	0	0	0	0	1	(2)		
1.07–1.22 m^2^	0	0	2	(2)	3	(5)		
1.23–1.37 m^2^	3	(2)	15	(12)	16	(29)		
1.38–1.52 m^2^	19	(15)	45	(35)	15	(27)		
1.53–1.68 m^2^	40	(32)	40	(31)	13	(24)		
1.69–1.83 m^2^	38	(31)	21	(16)	7	(13)		
1.84–1.98 m^2^	13	(10)	5	(4)	0	0		
1.99–2.14 m^2^	11	(9)	1	(1)	0	0		
≥ 2.15 m^2^	0	0	1	(1)	0	0		
Albumin								
Median (range), g/dL	3.7	(1.4–4.9)	3.6	(0.5–4.8)	3.75	(2.3–4.5)	0.34*	0.98*
Primary tumor location							0.10	0.17
Right	17	(14)	28	(22)	12	(22)		
Left	107	(86)	102	(78)	43	(78)		
Histological grade							0.02	0.09
Well and moderately differentiated adenocarcinoma	106	(86)	121	(93)	53	(96)		
Other adenocarcinoma	14	(11)	3	(2)	1	(2)		
Unknown	4	(3)	6	(5)	1	(2)		
RAS status							0.20	0.28
* RAS/KRAS* wild type	69	(56)	59	(46)	26	(47)		
* RAS/KRAS* mutant type	54	(43)	68	(52)	27	(49)		
Unknown	1	(1)	3	(2)	2	(4)		
Metastatic organ site								
Liver	84	(68)	78	(60)	26	(47)	0.20	0.009
Lung	84	(68)	87	(67)	36	(66)	0.89	0.76
Lymph node	56	(45)	53	(41)	25	(46)	0.48	0.97
Peritoneum	22	(18)	26	(20)	15	(27)	0.65	0.15
Bone	21	(17)	11	(9)	8	(15)	0.04	0.69
Local	5	(4)	9	(7)	6	(11)	0.31	0.08
Number of metastatic organ sites							0.22	0.69
1	25	(20)	33	(25)	14	(26)		
2	45	(36)	54	(42)	20	(36)		
≥ 3	54	(44)	43	(33)	21	(38)		
Duration from initiation of first-line chemotherapy							0.71	0.37
≥ 18 months	89	(72)	96	(74)	43	(78)		
Prior regimens							0.99	0.59
≥ 3	64	(52)	67	(52)	26	(47)		
CEA							0.26	0.65
≥ 5.0 mg/dL	113	(91)	110	(85)	52	(95)		
Missing	1	(1)	3	(2)	0	(0)		

*Mann–Whitney test.

ECOG PS, Eastern Cooperative Oncology Group performance status; BSA, body surface area; CEA, carcinoembryonic antigen.

### Incidences of severe AEs according to renal impairment

Grade 3 or higher AEs according to the renal impairment group are shown in Table 2. In terms of hematologic AEs, neutropenia and anemia were 53% and 20%, respectively, in the moderate-to-severe group compared to the normal group (24%, *P* < 0.001 and 7%, *P* = 0.007, respectively). Regarding non-hematologic AEs, the incidence of febrile neutropenia was higher in the moderate-to-severe group (7%) than in the normal group (1%; *P* = 0.014). There were no significantly higher incidences of AEs in the mild group than in the normal group.

**Table 2:** Grade ≥ 3 adverse events according to the severity of creatinine clearance.

	Normal		Mild		Moderate to severe		*P* value (vs normal)	
	n	%	n	%	n	%	Mild	Moderate to severe
Any hematologic AE	38	(31)	53	(41)	31	(56)	0.09	0.001
Neutropenia	30	(24)	44	(34)	29	(53)	0.09	< 0.001
Anemia	8	(7)	14	(11)	11	(20)	0.22	0.007
Thrombocytopenia	4	(3)	4	(3)	1	(2)	0.95	0.6
Any nonhematologic AE	12	(10)	17	(13)	11	(20)	0.39	0.057
Fatigue	3	(2)	2	(2)	2	(3)	0.61	0.65
Anorexia	6	(5)	7	(5)	5	(9)	0.84	0.27
Diarrhea	1	(1)	1	(1)	1	(2)	0.97	0.55
Stomatitis	0	(0)	0	(0)	0	(0)	NA	NA
Infection	0	(0)	0	(0)	0	(0)	NA	NA
Febrile Neutropenia	1	(1)	4	(3)	4	(7)	0.19	0.015
Nausea	0	(0)	1	(1)	0	(0)	0.33	NA
Liver dysfunction	1	(1)	0	(0)	0	(0)	0.31	0.5
Interstitial pneumonia	0	(0)	0	(0)	0	(0)	NA	NA
Skin disorders	0	(0)	0	(0)	1	(2)	NA	0.13

AE, adverse event; NA, not available.

### Severity grade of renal impairment-induced AE

Renal impairment as a risk factor for the onset of grade 3 or higher AEs is shown in Table 3 and the ORs of the adjusted factors are shown in Supplementary Tables S1 and S2. Moderate-to-severe renal impairment was a significant risk factor for any grade 3 or higher hematologic AEs (adjusted OR 2.60; 95% CI 1.12–6.05; *P* = 0.026), especially in grade 3 or higher neutropenia (adjusted OR 3.47; 95% CI 1.45–8.30; *P* = 0.005). While moderate-to-severe renal impairment was not a significant risk factor of any grade 3 or higher non-hematologic AEs (adjusted OR 3.22; 95% CI 0.97–10.64; *P* = 0.056), no specific type of AEs was observed. Mild renal impairment was not a significant risk factor for hematologic or non-hematologic AEs.

**Table 3:** Logistic regression analyses for the onset of adverse events.

Grade ≥ 3 adverse event	Ccr group	Adjusted OR	95% CI	*P* value
Any hematologic adverse events	None	1		
	Mild	1.48	(0.80–2.76)	0.215
	Moderate and severe	2.6	(1.12–6.05)	0.026
Neutropenia	None	1		
	Mild	1.64	(0.85–3.15)	0.14
	Moderate and severe	3.47	(1.45–8.30)	0.005
Anemia	None	1		
	Mild	1.2	(0.40–3.64)	0.748
	Moderate and severe	1.79	(0.47–6.84)	0.395
Thrombocytopenia	None	1		
	Mild	0.48	(0.09–2.65)	0.4
	Moderate and severe	0.18	(0.01–2.88)	0.227
Any non-hematologic adverse events	None	1		
	Mild	1.8	(0.70–4.61)	0.222
	Moderate and severe	3.22	(0.97–10.64)	0.056
Fatigue/malaise	None	1		
	Mild	1.42	(0.16–12.92)	0.759
	Moderate and severe	5.08	(0.33–78.67)	0.245
Anorexia	None	1		
	Mild	1.56	(0.39–6.16)	0.533
	Moderate and severe	2.73	(0.48–15.49)	0.256
Diarrhea	None	1		
	Mild	0.29	(0.00–181.99)	0.709
	Moderate and severe	0.11	(0.00–224.23)	0.572
Febrile neutropenia	None	1		
	Mild	3.8	(0.27–35.22)	0.365
	Moderate and severe	5.69	(0.33–97.55)	0.231

Ccr, creatinine clearance; OR, odds ratio.

### Efficacy according to renal impairment

**Figure 2:**
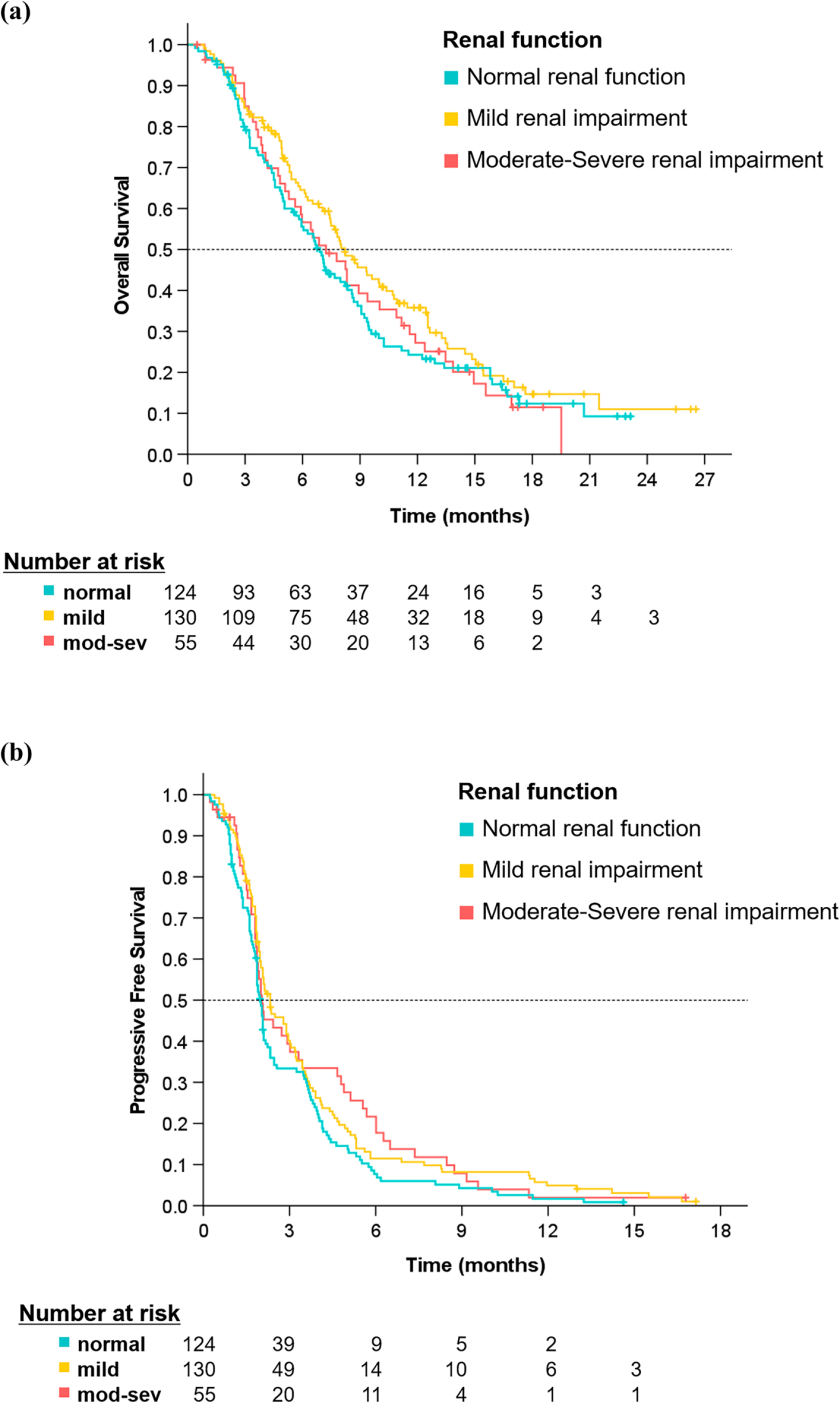
Kaplan–Meier curves of overall survival (**a**) and progression-free survival (**b**) according to renal impairment.

Two hundred thirty-two patients (75.1%) died, and disease progression was observed in 292 patients (94.5%), with a median follow-up time of 17.2 months. The Kaplan–Meier curves for OS and PFS according to renal impairment are shown in Fig. 2. The median OS was 6.9 months (95% CI 5.9–7.9) in the normal group, 8.2 months (95% CI 6.7–9.7) in the mild group, and 7.2 months (95% CI 5.2–9.3) in the moderate-to-severe group, indicating no significant difference between the normal group and the mild and moderate-to-severe groups (HR 1.3; 95% CI 0.9–1.7; *P* = 0.13 and HR 1.0; 95% CI 0.7–1.5; *P* = 0.88, respectively). Median PFS was 2.0 months (95% CI 1.8–2.1) in the normal group, 2.3 months (95% CI 1.7–2.9) in the mild group, and 2.0 months (95% CI 1.5–2.6) in the moderate-to-severe group, indicating no significant difference between the normal group and the mild and the moderate-to-severe groups (HR 1.3; 95% CI 1.0–1.6; *P* = 0.07 and HR 1.3; 95% CI 0.9–1.8; *P* = 0.13, respectively).

## Discussion

We found that moderate-to-severe renal impairment was a significant risk factor for the onset of severe neutropenia in patients treated with FTD/TPI, but the efficacy outcomes were not affected. Severe hematologic AEs were more likely to develop in the moderate-to-severe group; in particular, neutropenia. Previous reports have indicated that hematologic AEs, including neutropenia and anemia, are common in patients who receive FTD/TPI^1,4,7^. Although anemia was not a significant risk factor for renal impairment in our study, the incidence of severe anemia was significantly higher in the moderate-to-severe group than in the normal group (20% vs. 7%). Similar results have been reported, where the incidence of anemia of grade 3 or higher in patients with moderate or severe renal impairment numerically increased compared to that in patients with normal renal function or mild renal impairment (37–42% vs. 13–14%)^4^. In a phase III study, FTD/TPI was recognized as later-line standard chemotherapy for advanced gastric cancer (TAGS)^8^. Recently, the results of pooled safety analysis from the TAGS and RECOURSE studies have been reported^9^. The incidence of grade 3 or higher hematologic AEs, particularly neutropenia and anemia, was higher in patients with moderate renal impairment than in those with normal renal function. No correlation has been reported between non-hematologic AEs and renal impairment^4,9^. Our study showed that moderate-to-severe renal impairment may be a risk factor for severe non-hematologic AEs (*P* = 0.056). Unfortunately, specific AEs were not observed; however, the incidence of febrile neutropenia was higher in the moderate-severe group than in the normal group. In addition, this renal impairment may be a potential risk factor for severe anorexia (adjusted OR 2.73; *P* = 0.256) and fatigue (adjusted OR 5.08; *P* = 0.245). Even if these AEs are mild, they have a negative impact on quality of life. We should pay attention to these AEs when administering FTD/TPI to such patients.

On subgroup analysis of the TAGS data, grade ≥ 3 neutropenia was found to be more common in older patients aged ≥ 65 and ≥ 75 years than in younger patients aged < 65 years^10^. Generally, the risk of renal impairment increases with age. Our results showed that older age was common in patients with renal impairment; however, age was not a risk factor for AEs. Thus, before administering FTD/TPI in future clinical trials or daily practice setting, renal function should be evaluated using the Ccr value, regardless of age, and not based on serum creatinine level of < 1.5 mL/dL, which has been adopted in previous clinical trials^1,7^.

The OS and PFS of patients with renal impairment were comparable with those of patients with normal renal function. Therefore, FTD/TPI can be a candidate drug, even for patients with renal impairment; however, attention must be paid to the risk of the onset of severe AEs. In contrast, a previous report showed that the early onset of neutropenia predicted an improvement in OS in patients treated with FTD/TPI^11^. Therefore, the large area under the plasma concentration for FTD was associated with a significantly increased risk of neutropenia. Considering that FTD is metabolized by the liver, neutropenia may not be a useful predictive marker for patients with renal impairment.

Our study had limitations. We did not evaluate mild AEs of grades 1 and 2, which might have revealed additional significant risk factors if included. Collecting data on the time to AE onset could have been valuable, as early AEs might impact treatment continuity. Given the small sample size, specific types of AEs could not be identified. All patients included in this study were Japanese. In the Japanese population, it has been shown that Ccr based on the estimated glomerular filtration rate better reflects renal function than that based on the Cockcroft–Gault formula^12^. Thus, future studies should calculate the Ccr values using different methods.

## Conclusions

Moderate-to-severe renal impairment was found to be a risk factor for severe hematologic or non-hematologic AEs, particularly neutropenia, in mCRC patients treated with FTD/TPI; however, the efficacy outcomes were not affected. Therefore, we should be careful when administering the drug to this patient population.

### Supplementary Information

Supplementary Table S1.

Supplementary Table S2.

## Data Availability

The datasets used and/or analyzed during the current study available from the corresponding author on reasonable request.
