# Supplementary material for: Renal impairment as a risk factor for trifluridine/tipiracil-induced adverse events in metastatic colorectal cancer patients from the REGOTAS study
Source: Sci Rep. 2023 Oct 20;13:17931. doi: 10.1038/s41598-023-45244-7 (PMC10589204; doi:10.1038/s41598-023-45244-7)
Supplement: Supplementary file 1 — Supplementary Table S1. [file 41598_2023_45244_MOESM1_ESM.docx]

| **Table S1.** Logistic regression analyses for the onset of grade ≥3 hematologic adverse events | | | | | | | | | | | | | |
| --- | --- | --- | --- | --- | --- | --- | --- | --- | --- | --- | --- | --- | --- |
|  |  | Any hematologic AE | | | Neutropenia | | | Anemia | | | Thrombocytopenia | | |
|  |  | OR | 95% CI | P value | OR | 95% CI | P value | OR | 95% CI | P value | OR | 95% CI | P value |
| Ccr group | |  |  |  |  |  |  |  |  |  |  |  |  |
|  | None | 1 |  |  | 1 |  |  | 1 |  |  | 1 |  |  |
|  | Mild | 1.48 | (0.80-2.76) | 0.215 | 1.64 | (0.85-3.15) | 0.14 | 1.2 | (0.40-3.64) | 0.748 | 0.48 | (0.09–2.65) | 0.4 |
|  | Moderate and severe | 2.6 | (1.12-6.05) | 0.026 | 3.47 | (1.45-8.30) | 0.005 | 1.79 | (0.47-6.84) | 0.395 | 0.18 | (0.01–2.88) | 0.227 |
| Age | |  |  |  |  |  |  |  |  |  |  |  |  |
|  | Younger | 1 |  |  | 1 |  |  | 1 |  |  | 1 |  |  |
|  | Older | 1.01 | (0.98-1.04) | 0.495 | 1.02 | (0.99-1.05) | 0.14 | 1 | (0.96-1.04) | 0.969 | 1.04 | (0.95–1.13) | 0.406 |
| BSA | |  |  |  |  |  |  |  |  |  |  |  |  |
|  | Smaller | 1 |  |  | 1 |  |  | 1 |  |  | 1 |  |  |
|  | Larger | 1.26 | (0.32-5.01) | 0.746 | 3.43 | (0.814-14.5) | 0.09 | 0.07 | (0.01-0.80) | 0.033 | 0.31 | (0.01–18.00) | 0.571 |
| Histologic type | |  |  |  |  |  |  |  |  |  |  |  |  |
|  | Unknown | 1 |  |  | 1 |  |  | 1 |  |  | 1 |  |  |
|  | Tub1+tub2 | 1.3 | (0.46–3.68) | 0.619 | 1.41 | (0.48-4.12) | 0.532 | 0.51 | (0.06-4.50) | 0.546 | NA |  |  |
|  | Other type | 2.27 | (0.66-7.86) | 0.195 | 0.88 | (0.22-3.53) | 0.855 | 4.81 | (1.05-22.07) | 0.043 | NA |  |  |
| Liver metastasis | |  |  |  |  |  |  |  |  |  |  |  |  |
|  | No | 1 |  |  | 1 |  |  | 1 |  |  | 1 |  |  |
|  | Yes | 0.73 | (0.44–1.20) | 0.21 | 0.8 | (0.47–1.34) | 0.392 | 0.75 | (0.34–1.67) | 0.482 | 0.83 | (0.19–3.68) | 0.807 |
| Peritoneal dissemination | | |  |  |  |  |  |  |  |  |  |  |  |
|  | No | 1 |  |  | 1 |  |  | 1 |  |  | 1 |  |  |
|  | Yes | 1.04 | (0.56-1.94) | 0.909 | 1.16 | (0.60-2.23) | 0.657 | 1.5 | (0.57–3.92) | 0.405 | 0.51 | (0.06–4.80) | 0.559 |
| Bone metastasis | |  |  |  |  |  |  |  |  |  |  |  |  |
|  | No | 1 |  |  | 1 |  |  | 1 |  |  | 1 |  |  |
|  | Yes | 1.22 | (0.60-2.51) | 0.582 | 1.36 | (0.64-2.87) | 0.417 | 1.86 | (0.64–5.39) | 0.253 | 1 | (0.11–9.04) | 0.997 |
| Local recurrence | |  |  |  |  |  |  |  |  |  |  |  |  |
|  | No | 1 |  |  | 1 |  |  | 1 |  |  | 1 |  |  |
|  | Yes | 1.65 | (0.64-4.30) | 0.303 | 0.89 | (0.32-2.44) | 0.818 | 5.38 | (1.77–16.40) | 0.003 | 4.57 | (0.75–27.77) | 0.099 |
