# Supplementary material for: Renal impairment as a risk factor for trifluridine/tipiracil-induced adverse events in metastatic colorectal cancer patients from the REGOTAS study
Source: Sci Rep. 2023 Oct 20;13:17931. doi: 10.1038/s41598-023-45244-7 (PMC10589204; doi:10.1038/s41598-023-45244-7)
Supplement: Supplementary file 2 — Supplementary Table S2. [file 41598_2023_45244_MOESM2_ESM.docx]

| **Table S2.** Logistic regression analyses for the onset of grade ≥3 non-hematologic adverse events | | | | | | | | | | |  | |  | | |  |  |  |  | |
| --- | --- | --- | --- | --- | --- | --- | --- | --- | --- | --- | --- | --- | --- | --- | --- | --- | --- | --- | --- | --- |
|  |  | Any non-hematologic AE | | | Fatigue | | | Anorexia | | | Diarrhea | | | Febrile neutropenia | | | | | | |
|  |  | OR | 95% CI | P value | OR | 95% CI | P value | OR | 95% CI | P value | OR | 95% CI | P value | OR | 95% CI | | | | P value |  |
| Ccr group | |  |  |  |  |  |  |  |  |  |  |  |  |  |  | | | |  |  |
|  | None | 1 |  |  | 1 |  |  | 1 |  |  | 1 |  |  | 1 |  | | | |  |  |
|  | Mild | 1.8 | (0.70-4.61) | 0.222 | 1.42 | (0.16-12.92) | 0.759 | 1.56 | (0.39-6.16) | 0.533 | 0.29 | (0.00-181.99) | 0.709 | 3.8 | (0.27-35.22) | | | | 0.365 |  |
|  | Moderate to severe | 3.22 | (0.97-10.64) | 0.056 | 5.08 | (0.33-78.67) | 0.245 | 2.73 | (0.48-15.49) | 0.256 | 0.11 | (0.00-224.23) | 0.572 | 5.69 | (0.33-97.55) | | | | 0.231 |  |
| Age | |  |  |  |  |  |  |  |  |  |  |  |  |  |  | | | |  |  |
|  | Younger | 1 |  |  | 1 |  |  | 1 |  |  | 1 |  |  | 1 |  | | | |  |  |
|  | Older | 0.99 | (0.96-1.03) | 0.68 | 0.954 | (0.88-1.03) | 0.25 | 0.973 | (0.92-1.03) | 0.324 | 1.41 | (0.92-2.17) |  | 1.04 | (0.94-1.14) | | | | 0.468 |  |
| BSA | |  |  |  |  |  |  |  |  |  |  |  |  |  |  | | | |  |  |
|  | Smaller | 1 |  |  | 1 |  |  | 1 |  |  | 1 |  |  | 1 |  | | | |  |  |
|  | Larger | 1.81 | (0.25-13.26) | 0.6 | 1.04 | (0.01-103.19) | 0.988 | 0.3 | (0.01-6.51) | 0.445 | NA | NA |  | 6.88 | (0.09-512.62) | | | | 0.381 |  |
| Histologic type | | |  |  |  |  |  |  |  |  |  |  |  |  |  | | | |  |  |
|  | Unknown | 1 |  |  | 1 |  |  | 1 |  |  | 1 |  |  | 1 |  | | | |  |  |
|  | Tub1+tub2 | 2.75 | (0.78-9.78) | 0.118 | 9.45 | (1.09-81.95) | 0.042 | 4.06 | (0.89-18.48) | 0.07 | NA | NA |  | 0 | NA | | | | 0.998 |  |
|  | Other type | 0.68 |  |  | NA | NA | NA | 1.46 | (0.16-13.75) | 0.739 | 0.03 | NA |  | 0 | NA | | | | 0.999 |  |
| Liver metastasis | | |  |  |  |  |  |  |  |  |  |  |  |  |  | | | |  |  |
|  | No | 1 |  |  | 1 |  |  | 1 |  |  | 1 |  |  | 1 |  | | | |  |  |
|  | Yes | 1.09 | (0.53-2.23) | 0.817 | 1.41 | (0.27-7.5) | 0.684 | 0.86 | (0.31-2.40) | 0.773 | NA | NA |  | 13.89 | (1.20-160.39) | | | | 0.035 |  |
| Peritoneal dissemination | | | |  |  |  |  |  |  |  |  |  |  |  |  | | | |  |  |
|  | No | 1 |  |  | 1 |  |  | 1 |  |  | 1 |  |  | 1 |  | | | |  |  |
|  | Yes | 1.76 | (0.79-3.89) | 0.166 | 2.33 | (0.43-12.59) | 0.328 | 1.98 | (0.68-5.77) | 0.209 | NA | NA |  | 3.7 | (0.72-19.05) | | | | 0.118 |  |
| Bone metastasis | | | |  |  |  |  |  |  |  |  |  |  |  |  | | | |  |  |
|  | No | 1 |  |  | 1 |  |  | 1 |  |  | 1 |  |  | 1 |  | | | |  |  |
|  | Yes | 0.74 | (0.24-2.34) | 0.615 | NA | NA | NA | 0.69 | (0.14-3.50) | 0.658 | NA | NA |  | 0 | NA | | | | 0.998 |  |
| Local recurrence | | | |  |  |  |  |  |  |  |  |  |  |  |  | | | |  |  |
|  | No | 1 |  |  | 1 |  |  | 1 |  |  | 1 |  |  | 1 |  | | | |  |  |
|  | Yes | 1.77 | (0.52-5.97) | 0.358 | 3.06 | (0.267-34.98) | 0.369 | 0.85 | (0.10-7.30) | 0.883 | 1.45 | (0.02-122.2) |  | 12.31 | (1.18-128.88) | | | | 0.036 |  |
